# Rethinking informed consent in the time of COVID-19: An exploratory survey

**DOI:** 10.3389/fmed.2022.995688

**Published:** 2022-09-27

**Authors:** Evelien De Sutter, Teodora Lalova-Spinks, Pascal Borry, Peggy Valcke, Els Kindt, Anastassia Negrouk, Griet Verhenneman, Jean-Jacques Derèze, Ruth Storme, Isabelle Huys

**Affiliations:** ^1^Clinical Pharmacology and Pharmacotherapy, Department of Pharmaceutical and Pharmacological Sciences, KU Leuven, Leuven, Belgium; ^2^Centre for IT and IP Law, KU Leuven, Leuven, Belgium; ^3^Centre for Biomedical Ethics and Law, Department of Public Health and Primary Care, KU Leuven, Leuven, Belgium; ^4^MyData-TRUST, Mons, Belgium; ^5^University Hospitals Leuven, Leuven, Belgium; ^6^Clinical Trial Center, University Hospitals Leuven, Leuven, Belgium; ^7^Ethics Committee Research, University Hospitals Leuven, Leuven, Belgium

**Keywords:** ethics, clinical research, pandemic (COVID-19), stakeholders, electronic informed consent, digital technology

## Abstract

**Background:**

Owing to the infectious nature of COVID-19, alternative solutions, such as electronic informed consent (eIC), needed to be implemented to inform research participants about study-related information and to obtain their consent. This study aimed to investigate stakeholders’ experiences with alternative consenting methods as well as their views on any regulatory or legal guidelines for eIC implementation in clinical research. Results may serve as the cornerstone to rethink the informed consent process in clinical research.

**Materials and methods:**

This study consisted of an online survey among three stakeholder groups across European Union (EU) Member States and the United Kingdom. The stakeholder groups included (i) investigators, (ii) data protection officers (DPOs) or legal experts working in the pharmaceutical industry, academia, and academic biobanks, and (iii) ethics committee (EC) members. Data collection occurred between April and December 2021. The data collected were analyzed using descriptive and inferential statistics.

**Results:**

The online survey was completed by 191 respondents, of whom 52% were investigators. Respondents were active in 24 out of the 27 EU Member States and the United Kingdom. The majority of each stakeholder group considered validated electronic methods moderately or extremely useful to re-consent previously enrolled research participants upon study amendments or to obtain consent from COVID-19 patients. Nevertheless, this exploratory survey identified that only 13% of DPOs/legal experts, 26% of investigators, and 41% of EC members had experience with eIC. In addition, results suggest that the legal acceptance of eIC across EU Member States and the United Kingdom is variable and that a definition of eIC, issued by national law or policy, is rarely available. The results also showed that the COVID-19 pandemic brought additional challenges to inform participants and to obtain their consent; for example, related to travel restrictions.

**Conclusion:**

A number of alternative consenting methods were recommended, for example by the European Medicines Agency, to ensure clinical study continuation during the COVID-19 pandemic. Although stakeholders support the use of eIC in clinical research, it seems that the experience with eIC is low. To harmonize eIC practices as much as possible, further investments in multi-stakeholder, multi-national guidance are needed.

## Introduction

The COVID-19 pandemic heavily affected the conduct of clinical research (1, 2). Ongoing clinical studies required protocol modifications to safeguard the wellbeing, safety, and rights of the research participants (3). For example, participants were not allowed to visit the research site to receive the investigational medicinal product (IMP). As a result, the distribution of the IMP shifted from the delivery at the research site to shipment to the participants’ home (4). In addition, to minimize physical contact between the research team and the research participants, alternative informed consent (IC) procedures were required. Next to the clinical studies that started during the pre-pandemic period, various new studies have been initiated; for example, to evaluate the efficacy of therapeutic medicines and vaccines against the Severe acute respiratory syndrome coronavirus 2 (1). These clinical studies also faced challenges to embody ethical principles such as informing participants and obtaining their IC for participation in the study. According to the Declaration of Helsinki, voluntary consent of participants, having the capacity of providing IC, must be obtained prior to their inclusion in medical research (5). Nevertheless, the decision-making capacity of COVID-19 patients may be impaired; for example, when experiencing respiratory distress and requiring ventilation (6).

To promote the safety of the research participants and the research team while maintaining the clinical research integrity, several guidance documents were issued (7). These documents, issued by the European Medicines Agency (EMA) and several national regulatory bodies, provided recommendations on the conduct of clinical studies during the COVID-19 pandemic (8). The guidance document of the EMA sets out alternative consent procedures to inform COVID-19 patients and to obtain their IC. For example, it could be considered to obtain these patients’ oral consent in the presence of an impartial witness (4). Due to urgent protocol modifications, mainly in clinical studies that started during the pre-pandemic period, there was a need to obtain re-consent of already included participants. Therefore, alternative means to re-consent were suggested. For example, the EMA states that “*any validated and secure electronic system already used in the trial in the particular member state for obtaining informed consent can be used as per usual practice and if in compliance with national legislation*” (4).

Electronic informed consent (eIC) is one of the digital alternatives that has been used during the COVID-19 pandemic to ensure clinical study continuation (9). According to the EMA, eIC refers to “*the use of any digital media (e.g., text, graphics, audio, video, podcasts, or websites) to firstly convey information related to the clinical trial to the trial participant and secondly document informed consent via an electronic device (e.g., mobile phones, tablets, or computers)*” (10). It establishes the opportunity for research participants to access the study-related information via a variety of interactive methods and to provide their eIC (11). Moreover, eIC may tailor the information based upon the participants’ needs and may engage them in a longitudinal relationship with the research team; for example, to receive study results (12). Despite the (potential) benefits of eIC, its adoption is slow due to various reasons, such as legal constraints or concerns about security and protection of personal data (11, 13, 14).

The effect of the COVID-19 pandemic on the conduct of clinical research has been immense and may result in new insights to improve the IC process (15, 16). However, the perspectives of stakeholders involved in clinical research on the use of alternative consenting methods during the COVID-19 pandemic is not yet investigated. In addition, empirical results related to stakeholders’ experience with eIC in clinical research are scarce. In an effort to address these gaps, this exploratory study provides an understanding into stakeholders’ views on obtaining IC before and during the COVID-19 pandemic as well as on how they experience the European or country-specific support, from a regulatory and legal point of view, for the implementation of eIC in clinical research. More concretely, the views of the following stakeholders were collected: (i) investigators, (ii) data protection officers (DPOs) or legal experts working in the pharmaceutical industry, academia, and academic biobanks, and (iii) members of ethics committees (ECs). The insights provided by these multiple stakeholder groups may be fundamental to capitalize on the evolving IC process in the context of future clinical research.

## Materials and methods

### Study design and data collection

This study consisted of an online survey that was disseminated to various stakeholder groups, involved in clinical research, across European Union (EU) Member States and the United Kingdom (see details below). This survey, made available in English via the Qualtrics platform, consisted of two main parts: (i) compliance with the General Data Protection Regulation (GDPR) for clinical studies and (ii) general aspects related to obtaining (electronic) IC. The results of the first part are described elsewhere (17). The second part of the survey consisted of (i) a general question aiming to investigate the best practices in IC communication, (ii) questions related to eIC to assess stakeholders’ experiences with and views on eIC, and more specifically, the regulatory and legal support related to its implementation, and (iii) questions to investigate stakeholders’ views on the alternative IC methods, recommended by for example the EMA, and the challenges in IC communication they may have experienced during the COVID-19 pandemic (Supplementary material). The survey included multiple choice questions, Likert scale questions, and open-ended questions to allow the collection of in-depth qualitative data. More concretely, a set of questions relevant across stakeholder groups as well as more tailored questions were included in the survey. The survey was pilot tested with a representative from each stakeholder group. During pilot testing, the representatives were asked to think aloud to facilitate the collection of spontaneous comments while filling out the survey (18). Based on their feedback, the survey was adapted to increase the understanding of questions. An online invitation to fill out the survey was distributed to stakeholders between April and December 2021.

### Participants and recruitment

The survey targeted the following stakeholder groups: (i) investigators (more specifically, we recruited physicians-investigators only), (ii) DPOs or legal experts working in the pharmaceutical industry, academia, and academic biobanks, and (iii) EC members. Stakeholders needed to be fluent in English, active in an EU Member State or the United Kingdom, and needed to be involved in clinical research. The survey was broadly disseminated to stakeholders. More concretely, dissemination occurred via social media (e.g., LinkedIn, Twitter), via the professional networks of the research team, and via international consortia and networks, such as the Innovative Medicines Initiative (IMI) Consortium Corona Accelerated Research & Development in Europe (CARE), the European Organization for Research and Treatment of Cancer (EORTC), the European Network of Research Ethics Committees (EUREC), the European Association of Health Law (EAHL), and the European Forum for Good Clinical Practice (EFGCP). The study was approved by the EC Research UZ/KU Leuven (S65106).

### Analysis

The results obtained were analyzed descriptively via Microsoft Excel. Percentages were calculated based on the number of respondents for each question. Due to the logic applied in the survey questions and respondent drop-out, the sample size varied throughout the survey. Additional inferential statistics to test for differences between stakeholder groups were performed using IBM^®^ SPSS^®^ Statistics 28.0. For these stakeholder analyses, the Fisher-Freeman-Halton Exact test was used to analyze categorical variables. A *p*-value of less than 0.05 was considered statistically significant.

## Results

### Stakeholder characteristics

Among 191 survey respondents, most were investigators (52%, *n* = 100/191), followed by EC members (24%, *n* = 46/191), and DPOs/legal experts (24%, *n* = 45/191). Respondents’ organizations were based in 24 out of the 27 EU Member States and the United Kingdom. A detailed overview of the country representation for each stakeholder group can be found in Table 1.

**TABLE 1:** Country representation for each stakeholder group.

Country	Investigators (*n* = 82) *n* (%)	DPOs/legal experts (*n* = 31) *n* (%)	EC members (*n* = 39) *n* (%)
Austria	2 (2%)	/	3 (8%)
Belgium	13 (16%)	8 (26%)	5 (13%)
Bulgaria	/	5 (16%)	/
Croatia	3 (4%)	/	1 (3%)
Czechia	2 (2%)	1 (3%)	/
Denmark	/	2 (7%)	/
Estonia	/	/	1 (3%)
Finland	/	/	2 (5%)
France	7 (9%)	2 (7%)	/
Germany	6 (7%)	3 (10%)	3 (8%)
Greece	/	/	3 (8%)
Ireland	1 (1%)	/	1 (3%)
Italy	14 (17%)	1 (3%)	/
Latvia	/	/	2 (5%)
Lithuania	3 (4%)	/	2 (5%)
Luxembourg	1 (1%)	/	1 (3%)
Malta	1 (1%)	/	/
The Netherlands	8 (10%)	1 (3%)	2 (5%)
Poland	1 (1%)	2 (7%)	2 (5%)
Portugal	5 (6%)	/	4 (10%)
Slovakia	1 (1%)	/	1 (3%)
Slovenia	2 (2%)	/	/
Spain	4 (5%)	/	2 (5%)
Sweden	1 (1%)	1 (3%)	/
United Kingdom	7 (9%)	5 (16%)	4 (10%)

The majority of EC members were physicians (33%, *n* = 13/39) and lawyers (26%, *n* = 10/39), and were involved in a local or regional EC (56%, *n* = 22/39). Over one-third of EC members reported to have another occupation, such as pharmacist or bioethicist. DPOs/legal experts were mainly working at a pharmaceutical company (19%, *n* = 6/31), a research institute (19%, *n* = 6/31) or other institutions/organizations (42%, *n* = 13/31) such as universities, consultancy companies or law firms (Table 2). In addition, the majority of DPOs/legal experts (71%, *n* = 22/31) was involved in multi-country clinical studies whereas investigators were mainly involved in national studies (71%, *n* = 58/82), more specifically in the country where they are based. Almost all EC members (90%, *n* = 35/39) had experience with assessing clinical studies conducted in several EU countries.

**TABLE 2:** Characteristics of EC members and DPOs/legal experts.

Characteristics	EC members (*n* = 39) *n* (%)
*Occupation*	
Physician	13 (33%)
Lawyer	10 (26%)
Patient representative	2 (5%)
Statistician	1 (3%)
Other (e.g., bioethicist, pharmacist)	13 (33%)
*Type of EC involved in*	
National EC	17 (44%)
Local or regional EC	22 (56%)
**Characteristics**	**DPOs/legal experts (*n* = 31)** ***n* (%)**
*Working environment*	
Academic sponsor of clinical trials	2 (7%)
Pharmaceutical company	6 (19%)
Clinical research organization	3 (10%)
Research institute	6 (19%)
Hospital	1 (3%)
Other	13 (42%)

**FIGURE 1:**
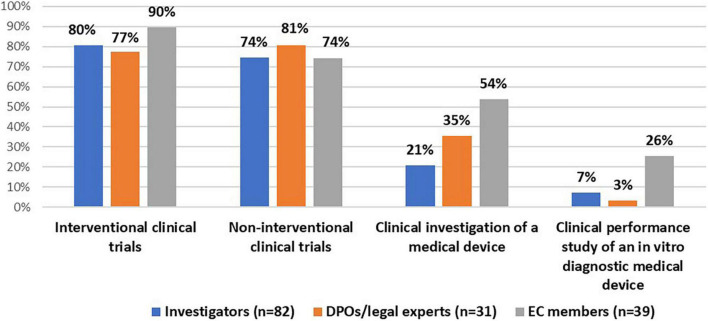
Stakeholders’ experiences with clinical studies.

**FIGURE 2:**
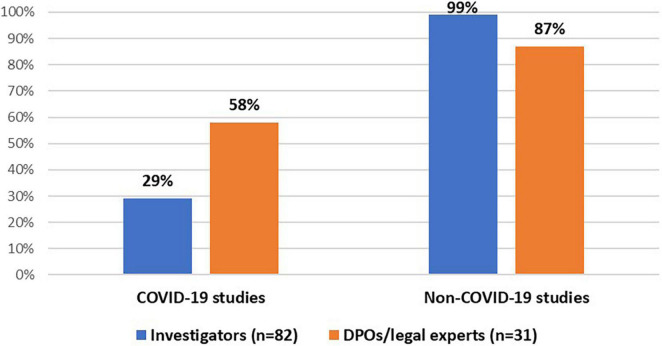
Stakeholders’ experiences with COVID-19 and non-COVID-19 studies.

All stakeholder groups were mainly involved in interventional and non-interventional clinical trials (Figure 1). Moreover, the majority of DPOs/legal experts (55%, *n* = 17/31) and investigators (61%, *n* = 50/82) were involved in the conduct of a registry-based trial. In addition, investigators (99%, *n* = 81/82) and DPOs/legal experts (87%, *n* = 27/31) were primarily involved in non-COVID-19 studies (i.e., clinical studies that investigate a medicine, diagnostic product or device, and/or vaccine for other medical conditions than COVID-19). In addition, some investigators (29%, *n* = 24/82) and the majority of DPOs/legal experts (58%, *n* = 18/31) had experience with COVID-19 studies (Figure 2).

### Best practices in informed consent communication

All stakeholders were asked what the ideal means of communication would be, regardless of the COVID-19 pandemic, to inform research participants about study-related information (e.g., the study objectives, study conduct, and risks and benefits). The 62 stakeholders who answered this open-ended question included 31 investigators, 12 DPOs/legal experts, and 19 EC members. All stakeholder groups emphasized the importance of in-person or virtual interactions between the research participants and the research team. It was raised that these personal interactions could support the provision of study-related information in an understandable and tailored manner. In addition, the majority of respondents across the stakeholder groups mentioned that this personal contact should be supported by written information, presented on paper-based forms or via electronic media. It was reported that research participants’ understanding may be facilitated by using flowcharts, illustrations or a video providing visual cues to point out important information. In addition, according to a limited number of EC members, a chat feature could be integrated in an eIC system that allows the research participants to ask questions to the research team or information could be offered in layers, enabling participants to access general and detailed information based on their preferences.

### Electronic informed consent

#### Experience

Only 13% (*n* = 2/15) of DPOs/legal experts and 26% (*n* = 11/43) of investigators had experience with informing research participants and obtaining their IC via electronic means. These two DPOs/legal experts indicated that their organizations used electronic means in interventional clinical trials, whereas only one organization had experience with the use of eIC in non-interventional clinical trials and in clinical investigations of a medical device. More specifically, both organizations made use of a tablet-enabled eIC while one organization conveyed information to participants and obtained their IC via a mobile phone. The majority of investigators had experience with eIC, in the format of a computer, in non-interventional clinical trials (Table 3). About 41% (*n* = 9/22) of EC members responded that their EC reviewed eIC in the past. The majority of them (75%, *n* = 6/8) considered the review process of eIC more complex than of paper-based IC forms. For example, it was considered essential to investigate whether the research participants’ personal data would be adequately protected when their IC is obtained electronically.

**TABLE 3:** Experience of investigators with eIC.

Types of clinical studies	Investigators (*n* = 11) *n* (%)
Interventional clinical trials	5 (46%)
Non-interventional clinical trials	10 (91%)
Clinical investigation of a medical device	1 (9%)
**Types of electronic means**	
Computer	9 (82%)
Tablet	4 (36%)
Phone	5 (46%)

#### Definition and functionalities

Stakeholders were asked whether they were aware of a definition of eIC, issued by national law or policy, in at least one of the countries where their organizations operate. Only three investigators (7%, *n* = 3/43), three DPOs/legal experts (20%, *n* = 3/15), and one EC member (5%, *n* = 1/22), all of their organizations operating in the United Kingdom, indicated that national law or policy provide a definition of eIC. In addition, an EC member active in Latvia mentioned that a new law on biobanks will be issued that will provide a definition of eIC. Finally, 67% (*n* = 29/43) of investigators, 40% (*n* = 6/15) of DPOs/legal experts, and 9% (*n* = 2/22) of EC members did not exactly know.

Survey respondents who indicated that they were not aware of a definition were asked what they understand by eIC. Based on the responses of all three stakeholder groups (i.e., 27 investigators, 9 DPOs/legal experts, and 17 EC members), three main viewpoints arose. The first viewpoint was that eIC refers to electronic means to convey information only whereas the second viewpoint consisted of electronic means to obtain and record the research participants’ consent only. The third viewpoint was that eIC enables research participants to review study-related information and to provide their consent electronically.

When asked for the type of functionalities that should be ideally part of an eIC system, almost all stakeholders indicated that it should enable the provision of study-related information in an interactive and dynamic way, followed by obtaining and documenting the participants’ signature and the possibility to re-consent participants upon study amendments (Table 4). Only for the latter functionality, a significant difference was found between stakeholder groups (*p* = 0,029) (Supplementary material). In addition, approximately half of investigators (52%, *n* = 22/42) and DPOs/legal experts (47%, *n* = 7/15), and a small majority of EC members (60%, *n* = 13/22) preferred to integrate the return of research results to the research participants in an eIC system. Other functionalities described by some stakeholders (11%, *n* = 9/79) related to the integration of a functionality that enables face-to-face contact between the research team and the participant, the possibility for the participants to withdraw their IC, the implementation of a quiz to test the participants’ understanding, and the possibility for the research team to invite research participants to participate in surveys.

**TABLE 4:** Type of functionalities of an eIC system.

Type of functionalities	Investigators (*n* = 42) *n* (%)	DPOs/legal experts (*n* = 15) *n* (%)	EC members (*n* = 22) *n* (%)
Providing research study information in an interactive and dynamic way to (potential) research participants	39 (93%)	14 (93%)	22 (100%)
Obtaining and documenting the signature of the research participants	36 (86%)	14 (93%)	18 (82%)
The possibility to re-consent research participants	29 (69%)	15 (100%)	18 (82%)
The return of research results to the research participants	22 (52%)	7 (47%)	13 (60%)
Other	1 (2%)	2 (13%)	6 (27%)

#### Legal acceptance

Stakeholders were asked whether it is legally allowed in at least one of the countries where their organization operates to provide study-related information to research participants via electronic means, before obtaining their IC. In total, 47% of investigators (*n* = 20/43), 40% of DPOs/legal experts (*n* = 6/15), and 59% of EC members (*n* = 13/22) stated that providing study-related information electronically is legally allowed. These stakeholders’ organizations were mainly operating in Belgium (10%, *n* = 4/39), Germany (10%, *n* = 4/39), Italy (10%, *n* = 4/39), Poland (10%, *n* = 4/39), and the United Kingdom (18%, *n* = 7/39). Nevertheless, some of the respondents (investigators: 5%, *n* = 2/43; DPOs/legal experts: 7%, *n* = 1/15; and EC members: 41%, *n* = 9/22) explicitly reported that providing information electronically can only take place under certain conditions. For example, there must be confirmation that the participant will also receive oral explanation about the clinical study or the participant must have the possibility to ask questions, if any. Additionally, 42% of investigators (*n* = 18/43), 53% of DPOs/legal experts (*n* = 8/15) and 23% of EC members (*n* = 5/22) did not know.

In a subsequent question, stakeholders were asked whether it is legally allowed in at least one of the countries where their organization operates to obtain research participants’ IC via electronic means. One-third of investigators (33%, *n* = 14/43) and the majority of DPOs/legal experts (60%, *n* = 9/15) and EC members (71%, *n* = 15/21) indicated that it is legally allowed whereas 56% of investigators (*n* = 24/43), 33% of DPOs/legal experts (*n* = 5/15) and 10% of EC members (*n* = 2/21) did not exactly know. The majority of stakeholders who stated that it is legally allowed were operating in Belgium (16%, *n* = 6/38), Germany (11%, *n* = 4/38), Poland (11%, *n* = 4/38), and the United Kingdom (26%, *n* = 10/38). Nevertheless, a few EC members (14%, *n* = 3/21) noted that the legal acceptance depends on the type of the clinical study. Stakeholders who indicated that it is legally allowed to obtain the research participants’ IC electronically were further asked to specify the type of signature(s) that are allowed to use. Generally, the majority of DPOs/legal experts (56%, *n* = 5/9) indicated that a simple or basic electronic signature as well as a qualified advanced electronic signature can be used (Table 5). It was mentioned that the type of suitable signature may depend on the risk level of the clinical study. Some DPOs/legal experts (22%, *n* = 2/9) and EC members (27%, *n* = 4/15) mentioned that other signatures are legally allowed to use, such as a tick box in combination with a check of the participants’ identity documents. Additionally, 50% of investigators (*n* = 7/14), 22% of DPOs/legal experts (*n* = 2/9), and 13% of EC members (*n* = 2/15) indicated that they did not know.

**TABLE 5:** Stakeholders’ views on the type of signatures that are legally allowed to obtain the research participants’ IC.

Type of signatures	Investigators (*n* = 14) *n* (%)	DPOs/legal experts (*n* = 9) *n* (%)	EC members (*n* = 15) *n* (%)
Simple or basic electronic signature	6 (43%)	5 (56%)	5 (33%)
Advanced electronic signature	4 (29%)	4 (44%)	3 (20%)
Qualified advanced electronic signature	1 (7%)	5 (56%)	6 (40%)
I do not know	7 (50%)	2 (22%)	2 (13%)
Other	0 (0%)	2 (22%)	4 (27%)

When asked which laws or regulations regulate (i.e., allow or prohibit) the use of eIC, respondents across stakeholder groups referred to EU regulations such as the Regulation on electronic identification and trust services for electronic transactions in the internal market (19), the GDPR (20), and the Clinical Trials Regulation (21). Other answers related to national legislation such as the Slovak Act No. 362/2011 on medicines and medical devices (22). Moreover, other respondents stated that there are no laws or regulations in place that specify the use of eIC in clinical research.

### Informed consent during the COVID-19 pandemic

#### Re-consenting already included research participants

Investigators and DPOs/legal experts were asked whether they used the following alternative methods to re-consent already included research participants during the COVID-19 pandemic, as recommended by for example the EMA: (i) obtaining oral consent (e.g., via phone or video-calls) supplemented with email confirmation, (ii) obtaining oral consent (e.g., via phone or video-calls) followed by an appropriately signed and dated IC as soon as possible, and (iii) using validated electronic systems (e.g., eIC). Most of investigators (41%, *n* = 17/42) and DPOs/legal experts (60%, *n* = 9/15) obtained the participant’s oral consent, followed by an appropriately signed and dated IC as soon as possible. Moreover, 19% of investigators (*n* = 8/42) and 33% of DPOs/legal experts (*n* = 5/15) made use of oral consent supplemented with email confirmation, and 17% of investigators (*n* = 7/42) and 13% of DPOs/legal experts (*n* = 2/15) used validated electronic systems (e.g., eIC) to re-consent for already included participants. Other methods used by 7% of investigators (*n* = 3/42) related to obtaining written IC in person.

**FIGURE 3:**
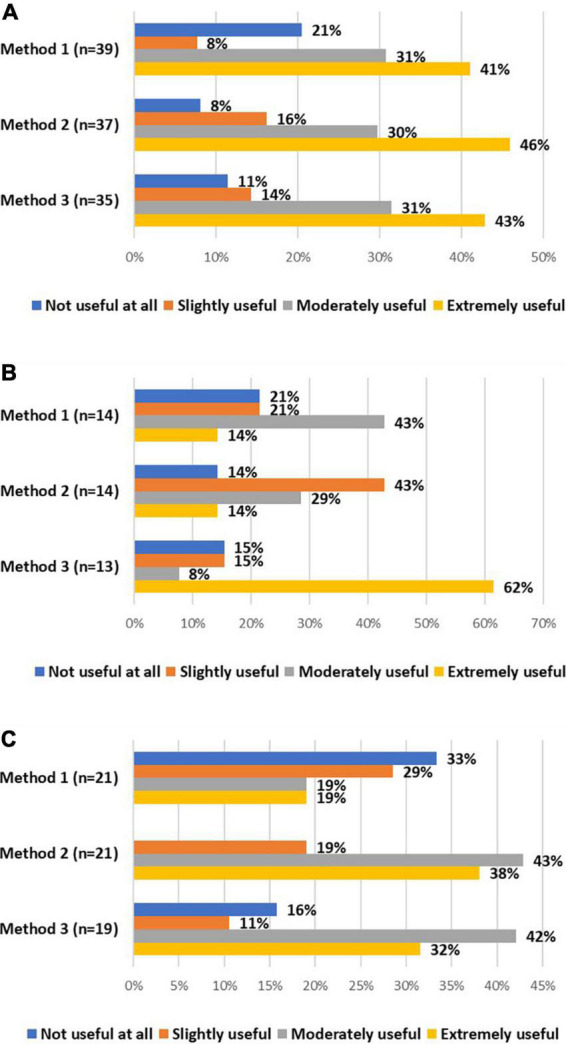
The views of investigators (A), DPOs/legal experts (B), and EC members (C) on the usefulness of method 1 (i.e., obtaining oral consent supplemented with email confirmation), method 2 (i.e., obtaining oral consent, followed by an appropriately signed and dated IC), and method 3 (i.e., using validated electronic systems) to re-consent for already included research participants.

All stakeholders were asked about the usefulness of these alternative method(s) to re-consent for already included research participants. The majority of investigators considered all alternative methods moderately or extremely useful (Figure 3A). On the contrary, the majority of DPOs/legal experts considered the second method slightly useful or not useful at all (Figure 3B). EC members mainly considered the second and third method moderately or extremely useful (Figure 3C). Potential advantages and challenges, raised by stakeholders, of these alternative methods can be found in Table 6.

**TABLE 6:** Potential advantages and challenges of alternative methods to re-consent for already included research participants.

**Obtaining oral consent, supplemented with email confirmation**	
**(Potential) advantages**	**(Potential) challenges**
• Saves participants to visit the research site • No pressure to provide research information during a medical consultation • Manageable	• Legality • Adequate email encryption to avoid a data breach • Identity check of participants • Traceability in case of audits and inspections
**Obtaining oral consent, followed by an appropriately signed and dated IC as soon as possible**	
**(Potential) advantages**	**(Potential) challenges**
• Saves participants to visit the research site • No pressure to provide research information during a medical consultation • Appropriate security and attributability	• Legality • Identity check of participants • Susceptible to documentation issues because of the risk of forgetting to obtain the participants’ written IC later on
**Using validated electronic systems**	
**(Potential) advantages**	**(Potential) challenges**
• Saves participants to visit the research site • No pressure to provide research information during a medical consultation • Unequivocal documentation and storage of signed consent forms • Information can be provided via multiple media	• Legality • Complex for participants who lack digital literacy • Access to electronic means • Numerous eIC systems are confusing • The setup may place time and logistical demands on the sponsor/study team

#### Obtaining informed consent from COVID-19 patients

Investigators and DPOs/legal experts were questioned which method(s) they used, if applicable, to obtain IC of COVID-19 patients. It should be noted that the responses were recorded between April and December 2021, when the COVID-19 pandemic continued its worldwide spread. In total, 41% of investigators (*n* = 16/39) and 64% of DPOs/legal experts (*n* = 9/14) did not have experience with obtaining IC of COVID-19 patients. Nevertheless, 31% of investigators (*n* = 12/39) and 29% of DPOs/legal experts (*n* = 4/14) indicated that the patients and the principal investigator signed and dated separate paper-based IC forms and that an appropriately signed and dated IC form was obtained later on. In addition, validated electronic methods were used by 26% of investigators (*n* = 10/39) and 7% of DPOs/legal experts (*n* = 1/14). A minority of investigators (10%, *n* = 4/39) and DPOs/legal experts (7%, *n* = 1/14) had experience with obtaining the patients’ oral IC in the presence of an impartial witness, who signed and dated the IC form. In addition, 8% of investigators (*n* = 3/39) indicated that they made use of other methods.

**FIGURE 4:**
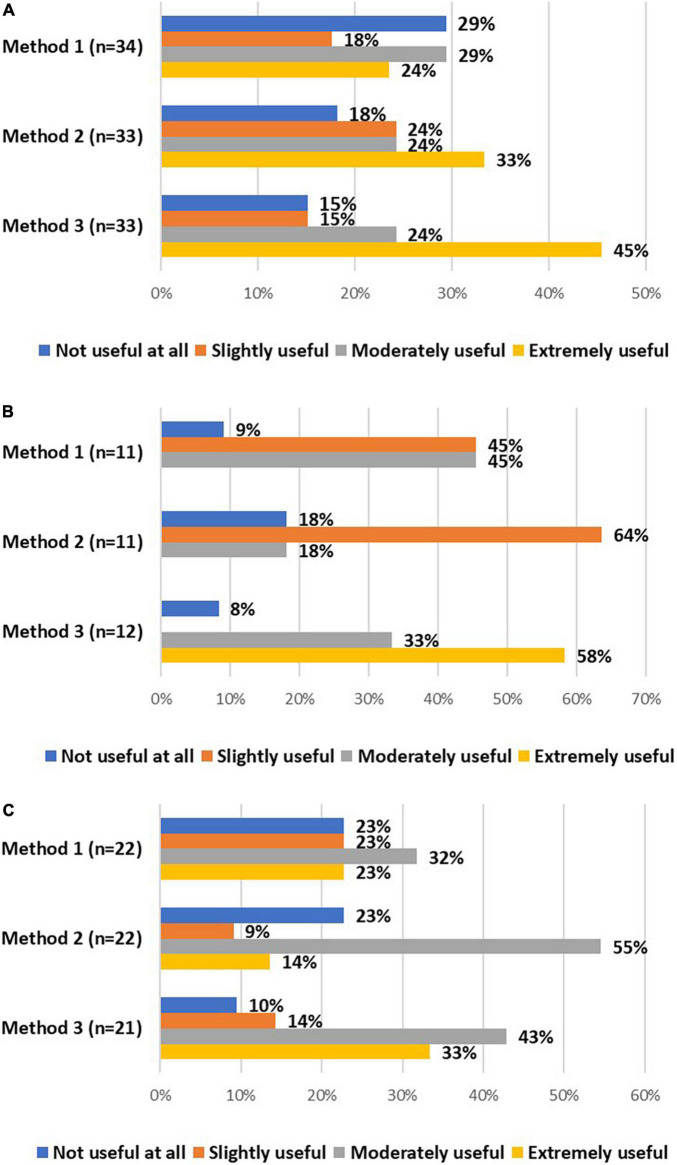
The views of investigators **(A)**, DPOs/legal experts **(B)**, and EC members **(C)** on the usefulness of method 1 (i.e., if written consent by the research participant is not possible, IC could be given orally by the research participant in the presence of an impartial witness, who is required to sign and date the IC form), method 2 (i.e., the research participant and the person obtaining consent sign and date separate IC forms and an appropriately signed and dated IC should be obtained from the participant as soon as possible), and method 3 (i.e., using validated electronic systems) to obtain IC from COVID-19 patients.

All stakeholders were asked about the usefulness of these methods. Investigators and DPOs/legal experts mainly considered using validated electronic methods moderately or extremely useful (Figures 4A,B). In addition, a vast majority of EC members considered signing and dating separate IC forms, followed by obtaining an appropriately signed and dated IC form later on, moderately or extremely useful, as well as using validated electronic systems (Figure 4C). The advantages and challenges of these methods, described by stakeholders, are displayed in Table 7.

**TABLE 7:** Potential advantages and challenges of alternative methods to obtain informed consent from COVID-19 patients.

**Obtaining oral consent in the presence of an impartial witness, who signs and dates the IC**	
**(Potential) advantages**	**(Potential) challenges**
• Trustworthy • Ensures impartiality	• Legality • Susceptible to documentation issues • Burdensome
**Signing and dating separate IC forms, followed by an appropriately signed and dated IC form as soon as possible**	
**(Potential) advantages**	**(Potential) challenges**
• Pragmatic solution	• Legality • Susceptible to documentation issues • Burdensome
**Using validated electronic systems**	
**(Potential) advantages**	**(Potential) challenges**
• Saves patients to visit the research site • Unequivocal documentation and storage of signed consent forms • Information can be provided via multiple media	• Legality • Complex for participants who lack digital literacy • Access to electronic means • Numerous eIC systems are confusing • The setup may place time and logistical demands on the sponsor/study team

### Challenges in informed consent communication prior to and during the pandemic

To evaluate the challenges in IC communication, investigators and DPOs/legal experts were asked about the challenges they experienced, prior to the COVID-19 pandemic, when providing study-related information to research participants and obtaining their IC. A common topic that arose from the responses of 12 investigators and 7 DPOs/legal experts was the provision of study-related information in understandable language and avoiding lengthy IC forms. Moreover, having enough time to convey detailed information about the study and the need to physically obtain IC were considered challenging by a few investigators. In addition, correct documentation of paper-based IC forms and deciding which type of consent (e.g., broad consent) is needed, were mentioned as other challenges by a limited number of DPOs/legal experts. The majority of investigators (74%, *n* = 26/35) and DPOs/legal experts (75%, *n* = 9/12) reported that they experienced the same challenges during the COVID-19 pandemic. A new challenge experienced, due to the COVID-19 pandemic, related to making sure that surfaces are disinfected to avoid COVID-19 transmission. In addition, the organization of face-to-face consultations was considered challenging. At particular moments during the pandemic, restrictions were in place to organize such consultations or study participants were not allowed to cross the border. When oral consent was obtained from the participant, it was raised that this consenting method impaired the ability to perceive the readability of emotions; for example, to check whether participants understood the study-related information. Also having proof of oral consent was considered challenging.

## Discussion

The arrival of COVID-19 has plummeted the clinical research activity in many countries. Multiple challenges were posed to ensure study continuation while minimizing the risk of infection for the participants as well as the research team; for example, related to informing research participants about study-related information and obtaining their IC (7). Because some alternative IC practices may be incorporated into common clinical practices in the future, it is key to understand stakeholders’ experiences and to investigate their opinions on the acceptance of eIC in the clinical research process.

### A need for guidance and harmonization of electronic informed consent

According to Mitchell et al., the use of eIC is increasing but is still relatively uncommon, which is in line with the results of our pilot survey (23). In addition, our survey found varying answers to some survey questions; for example, about the legal acceptance of eIC or about how stakeholders understand eIC. Given the complexity such divergence brings for the conduct of (especially multi-state) clinical trials, it may be argued that there is a substantial demand for more guidance about eIC in clinical research. Only a limited number of national regulatory bodies or ECs have issued public statements on eIC, such as Austria, Belgium, Denmark, and the United Kingdom (24–27). The European Commission, the Heads of Medicines Agencies and the EMA have launched the initiative “Accelerating Clinical Trials in the EU” (ACT EU) in 2022, which puts forward ten key priorities to facilitate the initiation, design, and conduct of clinical trials. In this ACT EU, the EU bodies state that “*disharmony of regulatory requirements between Member States complicates the submission of multi-state trial applications*” (28). In addition, the EMA’s “Regulatory Science to 2025” strategy indicates that the Good Clinical Practice (GCP) regulatory oversight can be modernized to enable the conduct of decentralized clinical trials, in which eIC can play an important role. The EMA’s strategy indicates that “*improving guidance on the design, conduct and analysis of clinical trials through broad stakeholder engagement, including patients and researchers can build a sound basis for advancing international consensus and its harmonization via organizations such as the International Council for Harmonisation of Technical Requirements for Pharmaceuticals for Human Use (ICH)*” (29). The ICH E6 GCP guideline is a cornerstone of clinical trial design, conduct, recording, and reporting (30). In March 2021, the ICH E6(R3) Expert Working Group published a draft of the updated ICH E6 principles. These updated principles are intended to support more efficient approaches to trial design, considering emerging technologies (31). Besides these initiatives, the EMA has already taken the necessary steps forward to set out unified requirements. In 2021, they issued a draft guidance on the use of computerized systems in clinical trials (10). Similarly, the US Food and Drug Administration issued a guidance specifically targeting eIC in clinical research in 2016 (32). Furthermore, one shall also keep in mind that reproducing evidence that patients have been duly informed and have provided a valid (electronic) consent is subject to national patient rights protection and evidence rules. Therefore, all these initiatives may contribute to clarifying the elements of valid consent and to harmonizing requirements related to the use of eIC in clinical research.

### Informed consent during the COVID-19 pandemic

During the COVID-19 pandemic, national and European regulatory bodies responded with guidance about the conduct of clinical trials during the outbreak. According to de Jong et al., 24 out of the 27 regulatory bodies of EU Member States issued such guidance, supplementary to the EMA guideline. More specifically, 16 guidances specifically addressed obtaining IC during the COVID-19 pandemic (33). Some included the use of electronic systems to obtain the participants’ IC, such as the guidances issued by the EMA and by the Belgian Federal Agency for Medicines and Health Products (4, 34). At the same time, in the Hungarian guidance it is stated that “*electronic patient information sheets and informed consent forms are not permitted, according to the law that must be followed in the current extraordinary situation as well*” (35). The group of the 27 national data protection authorities, known as the European Data Protection Board (EDPB), has also issued guidelines in which requirements for a valid consent under data protection law were discussed. The EDPB further stressed the need for a “freely given” consent in the context of the Clinical Trials Regulation (36, 37). Already before the COVID-19 pandemic, stakeholders supported the use of eIC as it offers opportunities compared to paper-based IC forms, such as using multimedia to convey information or enabling online storage of signed IC forms (11). Based on the survey data, it cannot be concluded whether the support for eIC has increased during the COVID-19 pandemic. Nevertheless, existing literature shows that the use of eIC in research centers located in the United States has increased during the pandemic, mainly for minimal-risk studies (38). Nevertheless, institutions without previous experience with eIC considered it challenging to educate a sufficient number of researchers to use eIC systems (39). Moreover, challenges were reported related to the accessibility of eIC for potential study participants, which is in line with the concerns raised by survey respondents (39).

Informing research participants about all the pertinent aspects of a clinical trial and obtaining their consent is a foundational ethical requirement (5). According to the available literature, the paper-based IC process may be ineffectual in truly informing research participants about the trial (12, 40–43). The changes in clinical practice, forced by the COVID-19 pandemic, offer an opportunity to reconsider the IC process in clinical research. As reported by survey respondents, eIC could offer particular advantages compared to the other recommended consenting methods. Similarly, Knoppers et al. stated that eIC can be used, including informational videos, to inform COVID-19 patients and to obtain their consent for the processing of their samples (44). Nevertheless, attention must be paid to the accessibility of this technology to participants as well as to a potential power imbalance (11, 37, 45). To this end, it could be advised to make use of an adaptive approach to the consent process, considering research participants’ needs and relationships, in order to maximize participant benefit.

### Study strengths and limitations

This study presents the views of key stakeholder groups involved in the set-up, review process or conduct of clinical studies, allowing us to gain a broad understanding of their experiences and views on informing research participants and obtaining their IC, before and during the COVID-19 pandemic. Moreover, these stakeholders represented 24 out of the 27 targeted EU Member States and the United Kingdom. This study aimed to equally represent these countries and the stakeholder groups involved. Nevertheless, it is important to recognize that investigators were the most responsive stakeholder group and that the majority of stakeholders were based in Belgium, Germany, Italy, the Netherlands, and the United Kingdom. The survey was widely disseminated via the network of research consortia and social media. Nevertheless, a small sample size and a high drop-out rate were observed. Potential reasons for this limitation may relate to survey fatigue, a lack of interest in the topic or a high workload due to the COVID-19 pandemic. Due to a small sample size, the generalization of survey results should be done with caution. Nevertheless, this exploratory nature of this survey provides valuable insights into stakeholders’ views on and experiences with alternative consenting methods, including eIC, as well as the main challenges they experience to inform participants and to obtain their consent, prior to and during the COVID-19 pandemic. Moreover, some questions remained theoretical to some survey respondents, for example, related to the usefulness of alternative consenting methods. Their views may change if they would gain practical experience with these methods.

## Conclusion

Our survey indicates that all stakeholder groups support the use of eIC during the COVID-19 pandemic and beyond. The survey data showed that the IC process, regardless of the COVID-19 pandemic, should ideally consist of personal interactions and written information. eIC offers the opportunity to convey information interactively, by making use of multimedia or by offering information in a layered approach. Nevertheless, stakeholders had little practical experience with eIC and reported some challenges that hinder the deployment of eIC in clinical research. In addition, it appears that there is a varying understanding of the term “eIC” as well as limited European or country-specific support, from a regulatory and legal point of view, to facilitate implementation. Therefore, multi-stakeholder, multi-national guidance could contribute to a more harmonized eIC approach in clinical research. Finally, the survey results showed that the COVID-19 pandemic resulted in some additional challenges to convey study-related information to participants and to obtain their consent.

## Data availability statement

The datasets presented in this article are not readily available because survey respondents did not provide their consent for the sharing of survey questionnaires with parties other than the researchers. The anonymized survey dataset can be made available to other researchers only upon request, ensuring that the information is not used for secondary data analysis without the prior consent of the research team who conducted this original study. Requests to access the datasets should be directed to EDS, Evelien.desutter@kuleuven.be.

## Ethics statement

The studies involving human participants were reviewed and approved by the Ethics Committee Research UZ/KU Leuven. The patients/participants provided their written informed consent to participate in this study.

## Author contributions

EDS, TL-S, and IH designed the study. The design of the study was further discussed with PV, EK, AN, GV, J-JD, and RS. EDS analyzed the data and drafted the manuscript. PB, PV, EK, AN, GV, J-JD, RS, and IH thoroughly reviewed the manuscript. All authors approved the final version of this manuscript.
