# Supplementary material for: Rethinking informed consent in the time of COVID-19: An exploratory survey
Source: Front Med (Lausanne). 2022 Sep 27;9:995688. doi: 10.3389/fmed.2022.995688 (PMC9552958; doi:10.3389/fmed.2022.995688)
Supplement: Supplementary file 1 [file Data_Sheet_1.DOCX]

Supplementary Material

Rethinking informed consent in the time of COVID-19: an exploratory survey

Evelien De Sutter^1*^, Teodora Lalova-Spinks^1,2^, Pascal Borry^3^, Peggy Valcke^2^, Els Kindt^2^, Anastassia Negrouk^4^, Griet Verhenneman^2,5^, Jean-Jacques Derèze^6^, Ruth Storme^7^, Isabelle Huys^1^

*** Correspondence:**Evelien De Sutter
Evelien.desutter@kuleuven.be

# Supplementary Materials

Supplementary material 1: Survey questionnaire

Supplementary material 2: Additional statistical analysis to assess differences between stakeholders

**Supplementary material 1: Survey questionnaire**

TOPIC 1: Introductory questions

# Which stakeholder group best characterizes you?

*Note: this question applies to all stakeholder groups*

I am a data protection officer or a member of a legal team (and I am not a member of an ethics committee)

I am a member of an ethics committee

I am an investigator/physician (and I am NOT a member of an ethics committee)

None of the listed stakeholder groups

# Where do you work? *(In case you work for more than one employer, please select the institution from the perspective of which you prefer to answer this survey)*

*Note: this question applies to data protection officers/legal experts only*

An academic sponsor of clinical trials

A pharmaceutical company

A biobank

A clinical research organization (CRO)

A research institute

A hospital

Other (please specify)

# What is your profession?

*Note: this question applies to ethics committee members only*

I am a physician

I am a lawyer

I am a patient representative

I am a study nurse

I am a statistician

I am a philosopher/priest

Other (please specify)

# Where is your organization based? *(Drop down list including European Union Member States and the United Kingdom)*

*Note: this question applies to all stakeholder groups*

# The ethics committee of which I am a member:

*Note: this question applies to ethics committee members only*

Is a national ethics committee

Is a local or regional ethics committee

# What types of clinical studies does the ethics committee of which you are a member assess? *(multiple answers possible)*

*Note: this question applies to ethics committee members only*

Clinical studies that are conducted only in the country where the ethics committee is based

Clinical studies that are conducted in several EU countries

# In which country is your organization involved/are you involved in clinical studies?

*Note: this question applies to data protection officers/legal experts and investigators only*

Only in the country where it is/I am based (national studies)

In several countries (international studies) (please specify in which countries)

# Please select the answer that is applicable to you. My organization/I was/am involved in: *(multiple answers possible)*

*Note: this question applies to data protection officers/legal experts and investigators only*

**COVID-19 studies**: clinical studies that investigate a COVID-19 medicine, diagnostic product or device, and/or vaccine

**Non-COVID-19 studies**: clinical studies that investigate a medicine, diagnostic product or device, and/or vaccine for other medical conditions (e.g., cancer)

# According to applicable legislation, there are different types of clinical studies. Below, we list several types of clinical studies. Please select the types of studies in which you are involved most often. *(multiple answers possible)*

*Note: this question applies to all stakeholder groups*

**Interventional clinical trials** *(Any investigation in human subjects intended to discover or verify the clinical, pharmacological and/or other pharmacodynamics effects of one or more investigational medicinal product(s), and/or to identify any adverse reactions to one or more investigational medicinal product(s) and/or to study absorption, distribution, metabolism and excretion of one or more investigational medicinal product(s) with the object of ascertaining its (their) safety and/or efficacy (Article 2(a) of Directive 2001/20/EC))*

**Non-interventional clinical trials** *(A study where the medicinal product(s) is (are) prescribed in the usual manner in accordance with the terms of the marketing authorisation. The assignment of the patient to a particular therapeutic strategy is not decided in advance by a trial protocol but falls within current practice and the prescription of the medicine is clearly separated from the decision to include the patient in the study. No additional diagnostic or monitoring procedures shall be applied to the patients and epidemiological methods shall be used for the analysis of collected data (Article 2(c) of Directive 2001/20/EC))*

**Clinical investigation of a medical device** *(A study in one or more human subjects, which serves the purpose of verifying or testing the safety and/or performance of a medical device.)*

**Clinical performance study of an in vitro diagnostic medical device** *(A study carried out to assess the clinical performance and safety of in vitro diagnostic medical devices.)*

# Has your organization/have you conducted a registry-based trial (i.e., an investigation of a research question which uses the infrastructure of a new or an existing registry for patient recruitment and/or data collection)?

*Note: this question applies to data protection officers/legal experts and investigators only*

Yes

No

TOPIC 2: General question related to informed consent

# What would be the ideal means of communication, regardless of the COVID-19 pandemic, to inform research participants about the objectives and conduct of a clinical study (e.g., study procedures, risks, benefits...)? *(Open question)*

*Note: this question applies to all stakeholder groups*

TOPIC 3: Electronic informed consent

# Does your organization/do you have experience with informing research participants and obtaining their informed consent via electronic means?

*Note: this question applies to data protection officers/legal experts and investigators only. Moreover, only if the answer is yes, the respondent is redirected to questions 13 and 14.*

Yes

No

I do not know

# For which clinical study(ies) does your organization/do you use electronic means to inform research participants and obtain their consent? *(multiple answers possible)*

*Note: this question applies to data protection officers/legal experts and investigators only*

**Interventional clinical trials** *(Any investigation in human subjects intended to discover or verify the clinical, pharmacological and/or other pharmacodynamics effects of one or more investigational medicinal product(s), and/or to identify any adverse reactions to one or more investigational medicinal product(s) and/or to study absorption, distribution, metabolism and excretion of one or more investigational medicinal product(s) with the object of ascertaining its (their) safety and/or efficacy (Article 2(a) of Directive 2001/20/EC))*

**Non-interventional clinical trials** *(A study where the medicinal product(s) is (are) prescribed in the usual manner in accordance with the terms of the marketing authorisation. The assignment of the patient to a particular therapeutic strategy is not decided in advance by a trial protocol but falls within current practice and the prescription of the medicine is clearly separated from the decision to include the patient in the study. No additional diagnostic or monitoring procedures shall be applied to the patients and epidemiological methods shall be used for the analysis of collected data (Article 2(c) of Directive 2001/20/EC))*

**Clinical investigation of a medical device** *(A study in one or more human subjects, which serves the purpose of verifying or testing the safety and/or performance of a medical device.)*

**Clinical performance study of an in vitro diagnostic medical device** *(A study carried out to assess the clinical performance and safety of in vitro diagnostic medical devices.)*

# Which electronic mean(s) does your organization/do you use to inform research participants and obtain their consent? *(multiple answers possible)*

*Note: this question applies to data protection officers/legal experts and investigators only*

Phone

Tablet

Computer

Other (please specify)

# Does national law or policy of the country or at least one of the countries where your organization operates/your ethics committee operates/you operate, provide a definition of electronic informed consent?

*Note: this question applies to all stakeholder groups. Moreover, only if the answer is “no” or “I do not know”, the respondent is redirected to question 16.*

Yes (please specify)

No

I do not know

# What do you understand by electronic informed consent? *(open question)*

*Note: this question applies to all stakeholder groups*

# Which functionalities should be part of an electronic informed consent platform (which is a platform enabling research participants to give and manage their electronic informed consent)? *(multiple answers possible)*

*Note: this question applies to all stakeholder groups*

Providing research study information in an interactive and dynamic way to (potential) research subjects

Obtaining and documenting the signature of the research subjects

The return of research results to the research subjects

The possibility to re-consent research subjects

Other (Please specify)

# Is it legally allowed in the country or in at least one of the countries where your organization operates/where your ethics committee is based/where you operate to provide study-related information to research participants via electronic means, before obtaining their informed consent?

*Note: this question applies to all stakeholder groups*

Yes

Only under certain conditions (please specify)

No

I do not know

# Is it legally allowed in the country or in at least one of the countries where your organization operates/where your ethics committee is based/where you operate to obtain research participants’ informed consent via electronic means?

*Note: this question applies to all stakeholder groups. Moreover, only if the answer is “Yes” or “Only under certain conditions”, the respondent is redirected to question 20.*

Yes

Only under certain conditions (please specify)

No

I do not know

# Which signature(s) is/are legally allowed to obtain the research participants’ informed consent? *(multiple answers possible)*

*Note: this question applies to all stakeholder groups.*

**Simple or basic electronic signature** *(data in electronic form which is attached to or logically associated with other data in electronic form and which is used by the signatory to sign)*

**Advanced electronic signature** *(an electronic signature which is (a) uniquely linked to the signatory, (b) is capable of identifying the signatory, (c) is created using electronic signature creation data that the signatory can, with a high level of confidence, use under his sole control and (d) is linked to the data signed therewith in such a way that any subsequent change is the data is detectable)*

**Qualified advanced electronic signature** *(advanced electronic signature that is created by a qualified electronic signature creation device, and which is based on a qualified certificate for electronic signatures)*

I do not know

Other (please specify)

# Which laws or regulations regulate (i.e., allow or prohibit) the use of electronic informed consent? (open question)

*Note: this question applies to all stakeholder groups*

# Did your ethics committee review electronic informed consent in the past?

*Note: this question applies to ethics committee members only. Moreover, only if the answer is “yes”, the respondent is redirected to question 23.*

Yes

No

I do not know

# In your experience, was the review process of electronic informed consent more complex, compared to the review process of paper-based informed consent?

*Note: this question applies to ethics committee members only*

Yes (please specify)

No

TOPIC 4: Informed consent during the COVID-19 pandemic

# Alternative methods were recommended, for example by the European Medicines Agency, to re-consent for already included research participants (e.g., due to protocol changes). These methods include:

**Method 1**: Obtaining oral consent (e.g., via phone or video-calls), supplemented with e-mail confirmation.

**Method 2**: Obtaining oral consent (e.g., via phone or video-calls). An appropriately signed and dated informed consent form should be obtained from the research participant as soon as possible.

**Method 3**: Using validated electronic systems (e.g., electronic informed consent).

Which method(s) did your organization/did you employ during the COVID-19 pandemic? *(multiple answers possible)*

*Note: this question applies to data protection officers/legal experts and investigators only*

Method 1

Method 2

Method 3

Other method (please specify)

Not applicable

# How useful are the alternative methods to re-consent for already included research participants?

*Note: this question applies to all stakeholder groups. Ethics committee members received more explanation on these alternative methods, as described in question 24. Moreover, if “not useful at all” or “slightly useful” is indicated, the respondent is redirected to question 26. If “moderately useful” or “extremely useful” is indicated, the respondent is directed to question 27.*

|  | Not useful at all | Slightly useful | Moderately useful | Extremely useful |
| --- | --- | --- | --- | --- |
| **Method 1** |  |  |  |  |
| **Method 2** |  |  |  |  |
| **Method 3** |  |  |  |  |

# Please specify why the recommended method(s) is/are not useful at all or slightly useful. *(open question)*

*Note: this question applies to all stakeholder groups.*

# Please specify why the recommended method(s) is/are moderately or extremely useful. *(open question)*

*Note: this question applies to all stakeholder groups.*

# Specific methods were recommended, for example by the European Medicines Agency, when obtaining informed consent of COVID-19 patients. These methods include:

**Method 1**: If written consent by the research participant is not possible, consent could be given orally by the research participant in the presence of an impartial witness. In such cases, the witness is required to sign and date the informed consent form and the investigator is expected to record how the impartial witness was selected.

**Method 2**: The research participant and the person obtaining consent sign and date separate informed consent forms (i.e., one informed consent form signed and dated by the participant and another consent form signed and dated by the researcher). An appropriately signed and dated informed consent form should be obtained from the research participant later, as soon as possible.

**Method 3**: Using validated electronic systems (e.g., electronic informed consent).

Which method(s) did your organization/did you employ during the COVID-19 pandemic? *(Multiple answers possible)*

*Note: this question applies to data protection officers/legal experts and investigators only*

Method 1

Method 2

Method 3

Other method (please specify)

Not applicable

# How useful are the alternative methods for obtaining informed consent of COVID-19 patients?

*Note: this question applies to all stakeholder groups. Ethics committee members received more explanation on these alternative methods, as described in question 28. Moreover, if “not useful at all” or “slightly useful” is indicated, the respondent is redirected to question 30. If “moderately useful” or “extremely useful” is indicated, the respondent is directed to question 31.*

|  | Not useful at all | Slightly useful | Moderately useful | Extremely useful |
| --- | --- | --- | --- | --- |
| **Method 1** |  |  |  |  |
| **Method 2** |  |  |  |  |
| **Method 3** |  |  |  |  |

# Please specify why the recommended method(s) is/are not useful at all or slightly useful. *(open question)*

*Note: this question applies to all stakeholder groups*

# Please specify why the recommended method(s) is/are moderately or extremely useful. *(open question)*

*Note: this question applies to all stakeholder groups*

# Prior to the pandemic, what where the biggest challenges that you experienced when providing research information to participants and obtaining their consent? *(open question)*

*Note: this question applies to data protection officers/legal experts and investigators only*

# During the pandemic, are these challenges the same, or different?

*Note: this question applies to data protection officers/legal experts and investigators only*

The same

Different (please specify)

TOPIC 5: Closing question

# Do you want to mention an additional point of concern regarding electronic informed consent, not covered in this survey? *(open question)*

*Note: this question applies to all stakeholder groups*

**Supplementary material 2: Additional statistical analysis to assess differences between stakeholders**

| **Question 17:** Which functionalities should be part of an electronic informed consent platform (which is a platform enabling research participants to give and manage their electronic informed consent)? | |
| --- | --- |
|  | **Fisher-Freeman-Halton Exact test: p-value** |
| **Functionality 1:**  Providing research study information in an interactive and dynamic way to (potential) research participants | p=0,510 |
| **Functionality 2:**  Obtaining and documenting the signature of the research participants | p=0,627 |
| **Functionality 3:**  The return of research results to the research participants | p=0,756 |
| **Functionality 4:**  The possibility to reconsent research participants | p=0,029 |
